# GSK3β mediates the carcinogenic effect of HPV16 in cervical cancer

**DOI:** 10.1038/srep16555

**Published:** 2015-11-12

**Authors:** Cuiling Ma, Chenglong Zeng, Liang Jin, Yang Yang, Pengfei Li, Liangfeng Chen, Jian Wang

**Affiliations:** 1 Department of Dermatology, Fourth Military Medical University, China; 2 Department of Obstetrics and Gynecology, Xijing Hospital, Fourth Military Medical University, China

## Abstract

Cervical cancer is one of the most prevalent and fatal cancers among women and infection of the human papillomavirus (HPV) is the most important risk factor. This study investigated how HPV16 regulated GSK3β expression and function to promote cervical cancers. The expression of GSK3β was analyzed by quantitative PCR and western blot. The proliferation, invasion, and clonogenic survival of cells with different E6/E7 levels were measured by MTT, transwell invasion assays, and soft agar colony-forming assays, respectively. The levels of GSK3β were correlated with the copy numbers and expression levels of HPV16 E6/E7 genes. HPV16 E6/E7 genes regulated GSK3β transcription through an element located in the promoter 85 and 250 base pairs upstream of the transcription start site. The abilities of cell proliferation, invasion, and clonogenic survival were increased in C33A cells by ectopic HPV16 E6/E7 and decreased in CaSki cells by knocking down HPV16 E6/E7 levels. Meanwhile, LiCl increased GSK3β transcript levels and the proliferation of CaSki cells in a HPV16-dependent manner. These data indicated that GSK3β may participated in HPV16 mediated deregulation of wnt/β-catenin and other signaling pathways promoting the progression and invasion of cervical cancers.

Cervical cancer is one of the most prevalent malignancies among women, especially in developing countries, with an estimated 530,000 new cases and 275,000 resultant deaths each year^1^. Persistent infection by high-risk human papillomavirus (HPV) is the underlying factor for the pathological progression to cervical cancer^1^,^2^. The early expressed oncoproteins E6 and E7 are the most important players of HPV infection and their levels could shape the type of HPV-mediated cervical lesion^3^. Oncogenic E6 protein physically associates with and promotes ubiquitin-dependent degradation of p53 proteins and other pro-apoptotic proteins^4^,^5^ whereas E7 binds to and deactivates retinoblastoma proteins (pRB)^5^,^6^, disrupting the normal cell cycle control and allowing neoplastic growth. Recent works indicate that the Wnt/β-catenin pathway could play a role in HPV16-mediated cervical cancer^7^.

The stabilization and nuclear translocation of cytosolic β-catenin is at the center of the canonical Wnt pathway. By forming complexes with transcription factors of the T-cell factor/lymphoid enhancing factor (TCF/LEF) family, nuclear β-catenin activates Wnt target genes such as c-Myc and cyclin D1^8^,^9^,^10^. In the absence of Wnt ligands, β-catenin is phosphorylated by a multiprotein “destruction complex” containing the tumor suppressor adenomatous polyposis coli (APC), the scaffold protein Axin, the glycogen synthase kinase 3β (GSK3β), and casein kinase 1. The phosphorylated β-catenin is ubiquitinated and targeted for degradation by the 26S proteasome. Upon secretion, Wnt glycoprotein binds to the N-terminal extracellular cysteine rich domain of its cell-surface receptors Frizzled (Fz) and the co-receptors of the low density lipoprotein receptor-related protein (LRP) family. Then the degradation of β-catenin by its destruction complex is stopped, which results in the nuclear accumulation of β-catenin and the activation of transcription of Wnt target genes^8^,^9^,^10^. The Wnt/β-catenin signaling pathway is deregulated in many types of cancers due to changes mostly in APC, Axin, or β-catenin and is suggested as an early event in tumorigenesis^8^,^9^.

GSK3β, one of the two isoforms of glycogen synthase kinase 3, is a multifunctional Ser/Thr kinase that regulates many downstream target genes and pathways^11^,^12^. As a component of the protein complex regulating the cellular level of β-catenin, no genetic change of GSK3β has been linked to the deregulation of β-catenin^8^,^9^,^10^ but GSK3β expression levels were increased in a variety of cancers^13^,^14^,^15^,^16^,^17^.

The β-catenin protein has been shown to be abnormally increased or localized in cervical cancer cells^18^,^19^,^20^, which has been associated with the progression of cervical cancer^7^,^19^,^20^. HPV oncoproteins have been shown to increase β-catenin accumulation by interfering with the β-catenin destruction complex or inhibiting its degradation by proteosome^21^,^22^,^23^,^24^. However, whether GSK3β plays a role in mediating HPV mediated perturbation of wnt/β-catenin in cervical cancer is not known. This study aimed to investigate whether GSK3β is a target of high risk HPV in cervical cancer.

## Results

### The GSK3β level and HPV copy number were correlated in cervical cancer cells

**Figure 1:**
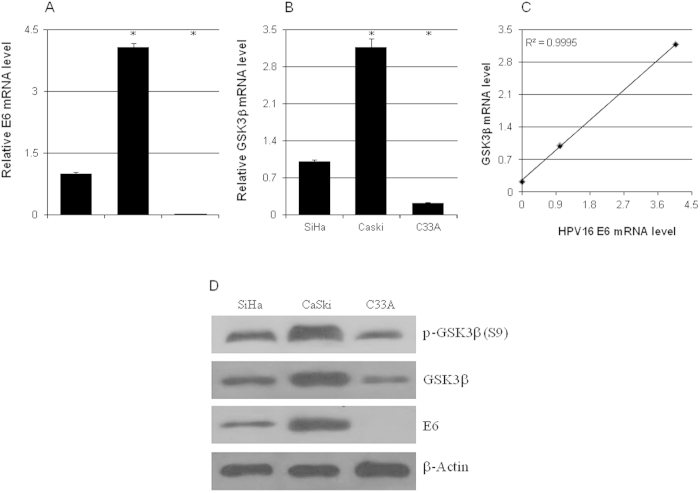
The expression levels of GSK3β and HPV16 E6 were correlated. The mRNA levels of HPV16 E6 (**a**) and GSK3β (**b**) in C33A, SiHa, and CaSki cells were analyzed by quantitative real-time PCR (n = 3). (**c**) The correlation between the mRNA levels of GSK3β and HPV16 E6. (**d**) Representitative western blot results of HPV16 E6, GSK3β, and phospho-GSK3β (S9). *p < 0.01 versus control.

To check if HPV infection had any effects on the expression level of GSK3β, its mRNA and protein levels in C33A (HPV negative), Siha (1~2 copies of HPV16), and CaSki (~600 copies of HPV16) were analyzed by real-time qPCR and western blot. CaSki cells had the highest level of GSK3β mRNA while C33A cells had significantly lower GSK3β mRNA level compared to Siha cells, which was highly correlated with the expression level of HPV16 E6 (Fig. 1A–C). The Serine 9 phosphorylated and total GSK3β protein levels were also correlated with the HPV16 E6 protein level in these cells (Fig. 1D).

### HPV16 oncoproteins E6/E7 upregulated GSK3β mRNA level in cervical cancer cells

**Figure 2:**
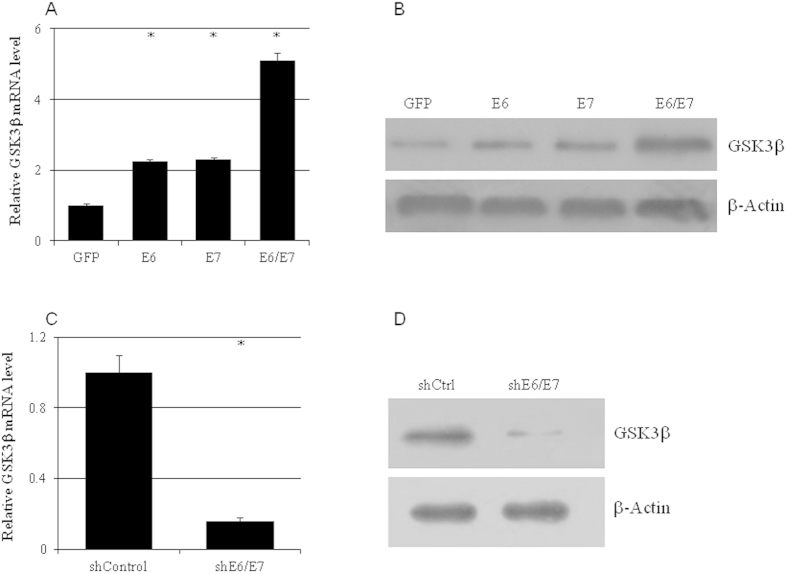
GSK3β expression was regulated by HPV16 E6/E7. The mRNA (**a,c**) and protein (**b,d**) levels of GSK3β in C33A overexpressing HPV16 E6 and/or E7 (**a,b**) or in CaSki cells treated with shRNA targeting HPV16 E6/E7 (**c,d**) (n = 3). *p < 0.01 versus control.

Ectopic expression and knockdown of HPV16 E6 and/or E7 were employed to investigate whether HPV16 oncoproteins E6/E7directly regulated the expression of GSK3β. The mRNA level of GSK3β in C33A cells was more than doubled with HPV16 E6 or E7 transfection and increased more than 5-fold with E6 and E7 double transfection (Fig. 2A). Accordingly, GSK3β protein exhibited a pattern similar to mRNA that was significantly increased by HPV16 E6 and E7 (Fig. 2B). On the other hand, knocking down HPV16 E6 and E7 in CaSki cells with shRNA significantly reduced the mRNA (Fig. 2C) and protein (Fig. 2D) levels of GSK3β.

### HPV16 E6/E7 increased the proliferation of cervical cancer cells

**Figure 3:**
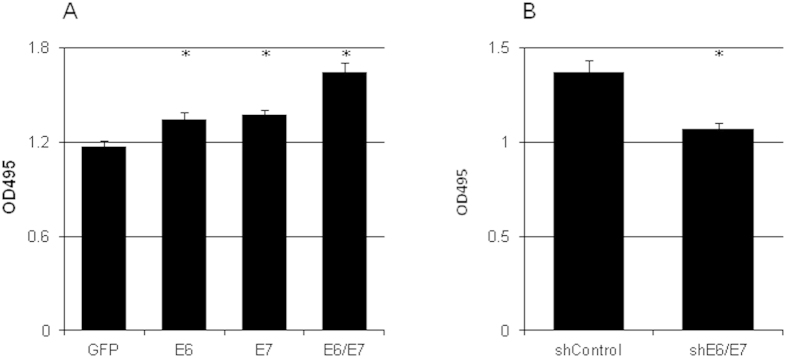
HPV16 E6/E7 controlled cervical cancer cell proliferation. The cell growth of C33A overexpressing HPV16 E6 and/or E7 (**a**) or CaSki cells treated with shRNA targeting HPV16 E6/E7 (**b**) was analyzed by MTT assay (n = 3). *p < 0.01 versus control.

The proliferation rate of C33A cells was increased 14.5%, 17.1%, and 40.2% by exogenous HPV16 E6, E7, and E6/E7, respectively (Fig. 3A). On the other hand, silencing HPV16 E6 and E7 in CaSki cells resulted in a 22% decrease of proliferation (Fig. 3B).

### HPV16 E6/E7 increased the metastatic ability of cervical cancer cells

**Figure 4:**
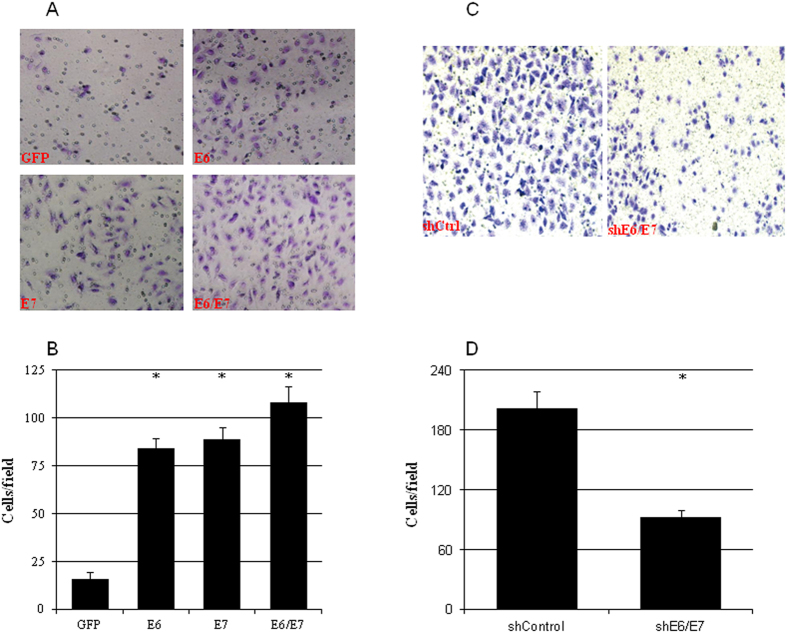
HPV16 E6/E7 enhanced the invading ability of cervical cancer cell. The number of invading cells was increased by ectopic expression of HPV16 E6, E7, and E6 and E7 in C33A cells (**a,b**) whereas it was decreased by knockdown HPV16 E6/E7 using shRNA in CaSki cells (**c,d**) (n = 3). *p < 0.01 versus control.

**Figure 5:**
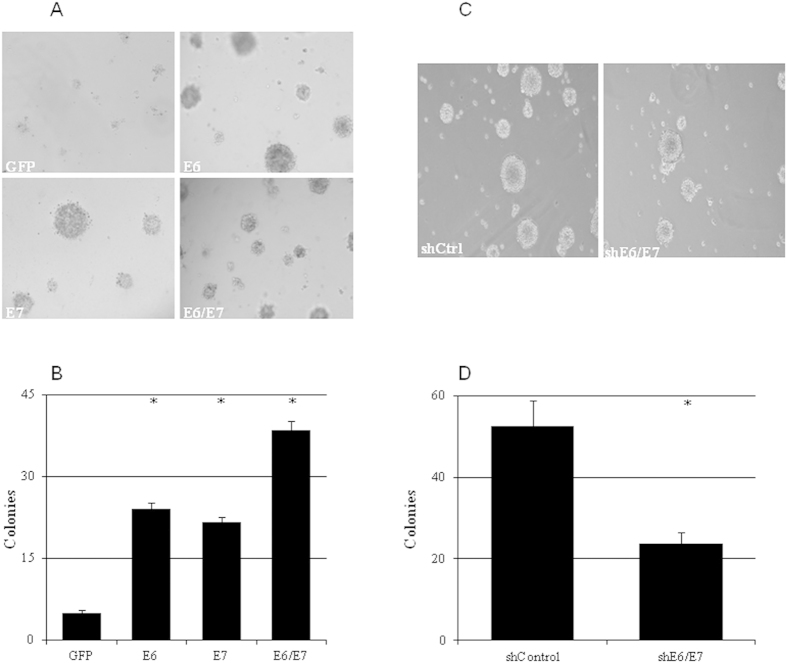
HPV16 E6/E7 enhanced the clonogenic survival of cervical cancer cell. The number of colonies was increased by ectopic expression of HPV16 E6, E7, and E6 and E7 in C33A cells (**a,b**) whereas it was reduced by shRNA knockdown of HPV16 E6/E7 using in CaSki cells (**c,d**) (n = 3). *p < 0.01 versus control.

The effects of HPV16 on the invading and colony-forming abilities of cervical cancer cells were evaluated by overexpressing HPV16 E6 and/or E7 in C33A cells or knocking down E6/E7 in CaSki cells. Exogenous HPV16 E6 or E7 significantly increased the numbers of invading cells (Fig. 4A,B) and colonies (Fig. 5A,B). Simultaneously overexpressing E6 and E7 further increased the numbers of invading cells (Fig. 4A,B) and colonies formed (Fig. 5A,B). On the other hand, silencing HPV16 E6/E7 expression in CaSki cells resulted in significant decrease of both invading cells (Fig. 4C,D) and colony formation (Fig. 5C,D).

### HPV16 E6/E7 were required for LiCl-induced increase of GSK3β expression

**Figure 6:**
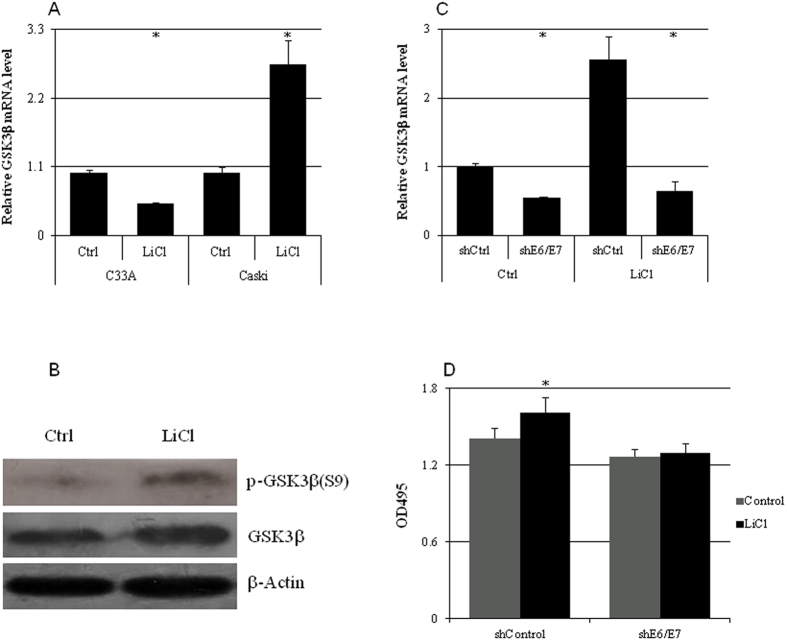
LiCl increased GSK3β expression and cell growth in a HPV16 E6/E7-dependent manner. (**a**) LiCl treatment for 24 hr resulted in significant increase of GSK3β mRNA level in CaSki cells but a reduction in C33A cells (n = 5). (**b**)The total GSK3β protein and phospho-GSK3β (S9) levels in CaSki cells were increased by 10 mM of LiCl treatment for 24 hr. (**c**) The GSK3β mRNA levels in CaSki cells infected with shcontrol or shE6/E7 lentivirues and treated with 10 mM of LiCl for 24 hrs (n = 5). (**d**) The proliferation of CaSki cells infected with shcontrol or shE6/E7 lentivirues and treated with 10 mM of LiCl for 24 hrs (n = 5). *p < 0.01 versus control.

The mRNA (Fig. 6A) and protein (Fig. 6B) levels of GSK3β in CaSki cells were significantly increased by LiCl (10 mM), which was abolished by E6/E7 knockdown (Fig. 6C). LiCl significantly increased the growth of CaSki cells which was also abrogated by E6/E7 knockdown(Fig. 6D).

### HPV16 E6/E7 regulated GSK3β transcription

**Figure 7:**
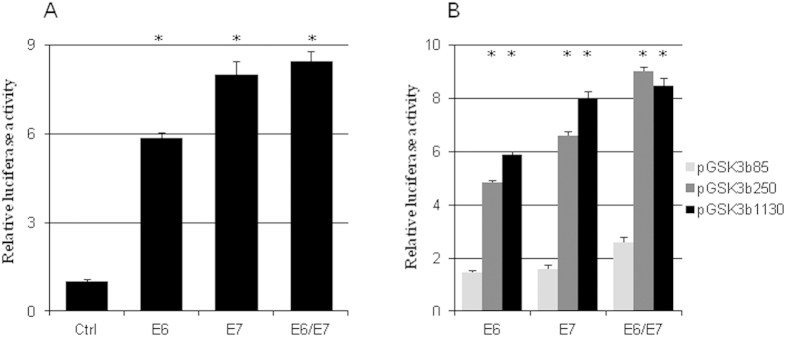
HPV16 E6/E7 regulated GSK3β expression on transcriptional level. (**a**) HPV16 E6, E7, and E6 and E7 significantly increased the luciferase activity of pGSK3β1130 (n = 5). (**b**) The luciferase activities of pGSK3β1130 and pGSK3β250 were significantly higher than that of pGSK3β85 in the presence of HPV16 E6 and/or E7 (n = 3). *p < 0.01 versus control in (**a**) or versus pGSK3β85 in (**b**).

Cotransfection of HPV16 E6 and/or E7 with the human GSK3β promoter luciferase reporter gene (pGSK3β1130) increased GSK3β promoter activity 5 to 9 fold (Fig. 7A). Deleting the promoter from −1130 bp to −250 bp did not significantly reduce human GSK3β promoter activity but further deleting the promoter to −85 bp resulted in the loss of a majority of the promoter activity (Fig. 7B).

## Discussion

The GSK3β expression including Ser9 phosphorylation was correlated with the HPV16 copy number and the expression of oncoprotein E6/E7. HPV16 E6/E7 regulated GSK3β transcription through a region between 85 bp and 250 bp upstream of the transcription initiation site of human GSK3β gene. Knocking down HPV16 E6/E7 reduced GSK3β expression in CaSki cells while overexpressing HPV16 E6 and/or E7 increased GSK3β expression in C33A cells. The proliferation, invading, and colony-forming abilities of cervical cancer cells were associated with HPV16 E6/E7 and GSK3β levels. A common GSK3β inhibitor LiCl increased GSK3β expression levels in CaSki cells, which was abolished by silencin HPV16 E6/E7 with shRNAs.

GSK3β serves as a master molecule for transducing various signals including Wnt and Notch to regulate cell cycle progression, cell differentiation, proliferation, and cell death^25^. In autochthonous transgenic prostate cancer TRAMP mice, inhibition of GSK3β resulted in reduction of tumor number and size through the derepression of C/EBPα and inhibition of E2F expression^16^. Inhibition of GSK3β by thiadiazolidinone (TDZD) in MM.1, U266, and other myeloma cells resulted in the inhibition of cell proliferation and induction of apoptosis via the dephosphorylation and activation of FOXO3a as well as increase of FasL and IκBα levels^13^. Similarly, GSK3β inhibition by AR-A014418 or siRNA in pancreatic cancer cell lines PANC-1 and MIA PaCa-2 reduced activation and nuclear accumulation of NFκB, which resulted in reduced cell growth and colony-formation^15^. However, whether the inhibition of GSK3β could sensitize pancreatic cancer to gemciatbine treatment was inconclusive^15^,^26^,^27^.

**Figure 8:**
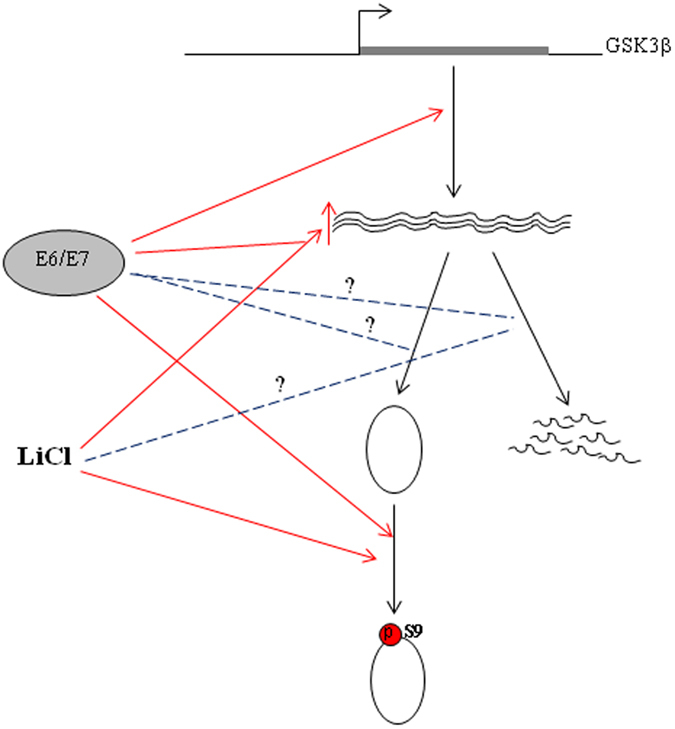
A proposed working model for HPV16 E6/E7 and LiCl regulating GSK3β in cervical cancers. HPV16 E6 and/or E7 up-regulate GSK3β transcription and may also play a role in GSK3β mRNA stability and translation. LiCl is known to increase the phosphorylation of GSK3β at Ser9 and increases GSK3β mRNA level in a HPV16 E6/E7-dependent manner in CaSki cells. 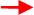 indicates the positive effects reported previously or in this report; 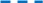 indicates the relationships that are postulated but have not been established.

This is the first study to investigate the relationship between high-risk HPV and GSK3β in cervical cancer. In cervical cancer cells, HPV16 E6/E7 promoted the expression of GSK3β at least in part through transcription. HPV16 E6 augmented the transcription activities of β-catenin responsive genes in HEK293 cells and cyclin D1 protein levels were correlated with HPV copy number in cervical cancer cells (C33A, SiHa, CaSki, and HeLa)^23^. In COS7 cells and primary human foreskin keratinocytes, HPV16 was shown to interact with DVL2 and β-catenin and enhanced the expression of c-myc and other β-catenin target genes^22^. HPV16 E6/E7 increased nuclear accumulation of β-catenin in oropharyngeal cancer cells by inhibiting the mediator of ubiquintination, Siah-1^21^. These results demonstrated that oncoprotein HPV E6/E7 regulated β-catenin protein level and nuclear accumulation at multiple levels and with different pathways. Our data presented a complicated picture regarding the effects of E6 and E7 oncoproteins of high-risk HPV on the regulation of GSK3β (Fig. 8), which in turn could modulate cellular β-catenin levels. HPV16 E6/E7 increased GSK3β transcripts by up-regulating GSK3β gene transcription, which resulted in the increase of steady-state GSK3β protein and Ser9 phosphorylated GSK3β protein levels.

Lithium chloride (LiCl) has been widely used to stimulate Wnt/β-catenin signal or regulate other pathways by deactivating the kinase activity of GSK3β^28^,^29^,^30^,^31^. LiCl treatment resulted in a significant increase of both mRNA and protein levels of GSK3β in CaSki cells, which was blunted by a knockdown of HPV16 E6/E7 with shRNA. The effect of LiCl on GSK3β was HPV16 E6/E7 dependent as LiCl was failed to increase GSK3β mRNA level in C33A cells. Moreover, as the increased level of GSK3β protein by HPV16 E6/E7 was in Ser9 phosphorylated state, the net effect would be the activation of wnt/β-catenin pathway^28^ or inflammation pathway^31^. As conventional wisdom believed that LiCl could only inhibit the kinase activity of GSK3β^28^,^29^,^30^,^31^, it was surprising that LiCl increased GSK3β mRNA level in HPV16 positive cervical cancer cells, which might have resulted from increased E6/E7 transcriptional activity by LiCl or GSK3β inactivation causing the activation of other signal pathway which cooperated with E6/E7. We are currently investigating the exact mechanisms underlying HPV16 E6/E7-dependent GSK3β mRNA level increase caused by LiCl.

The level of GSK3β expression correlated with copy numbers and expression levels of oncogenes of high-risk HPV in cervical cancer cell lines. The GSK3β level could be increased by ectopically expressing HPV16 E6/E7 in HPV negative C33A cells or reduced by knocking down E6/E7 level in HPV16 positive CaSki cells, which resulted in the changes in cell proliferation, invasion, and clonogenic survival. HPV16 E6/E7 up-regulated GSK3β transcription through a sequence element located between 85 and 250 bp upstream of the transcription initiation site. LiCl also increased GSK3β transcripts in an HPV16-dependent manner in cervical cancer cells.

## Methods

### Cell culture, transfection, and luciferase assay

C33A, Siha, CaSki, and 293T (ATCC, Manassas, VA) were routinely maintained in RPMI-1640 containing 10% FBS, 100 U/ml penicillin and 100 μg/ml streptomycin (all reagents from Life Technologies, Shanghai, China) at 37 °C in a humidified incubator with 5% CO_2_. Cells were split according to supplier’s recommendation when needed.

For LiCl treatment, C33A and CaSki cells were seeded into 6-well plates and cultured overnight before being treated with or without 10 mM LiCl for 24 hr. In the case of E6/E7 knockdown, CaSki cells were seeded into 6-well plates and infected with pLL3.7-shE6/pLL3.7-E7 or pLL3.7-shcontrol viruses for 36 hrs before treated with 10 mM LiCl for another 24 hrs. At the end of 24 hr LiCl treatment, cells were collected for MTT assay or RNA and protein works.

Transfection was done with lipofectamine 2000 (Life Technologies, Shanghai, China) according to manufacturer’s protocol. A luciferase activity assay was performed using the Dual-Glo^®^ Luciferase Assay System (Promega, Madison, WI) according to manufacturer’s instructions on a POLARstar Omega fluorescent reader (BMG Labtech, Germany).

### Quantitative real-time polymerase chain reaction (qPCR)

Total RNA was extracted from cells using Trizol reagent (Life Technologies) according to manufacturer’s instructions. The cDNA was synthesized from 1μg total RNA using SuperScript® III First-Strand Synthesis System (Life Technologies) and used as templates in ensuing qPCR reactions after 1:4 dilution with ddH_2_O. The qPCR was performed on an ABI 7300 (Applied Biosystems, Foster City, CA) using a qPCR kit from Transgen (Beijing, China) with the following protocol: 95 °C for 3 min followed by 40 cycles each of 95 °C for 30 sec, 55 °C for 30sec, and 70 °C for 30 sec. The oligonucleotide sequences of the primers were GCCAAGCCTTGTCAGAAATGA and TTTCTGGGCCATGGTTCTCT for GSK3β, TCAAAAGCCACTGTGTCCTG and CGTGTTCTTGATGATCTGCA for E6, ATTAAATGACAGCTCAGAGGA and GCTTTGTACGCACAACCGAAGC for E7, and GATGAGATTGGCATGGCTTT and GTCACCTTCACCGTTCCAGT for β-Actin. The relative gene expression level was calculated with the 2^−ΔΔCt^ method.

### Vector construction and virus production

The human GSK3β promoter reporter constructs were constructed by amplifying fragments 95bp (ATCGGTACCAGGACGAGTAGGAGG, pGSK3β85), 250bp (ATCGGTACCCGCAAACAAACGACGTC, pGSK3β250), and 1130bp (ATCGGTACCCTTGCAGCCGGCTGGGA, pGSK3β1130) upstream of the transcriptional start site with CGGAGATCTCTCCTCGCTTCCTTCCT as the 3′ primer from 293T genomic DNA and cloned into KpnI and BglII (TaKaRa Bio, Shiga, Japan) digested pGL3-basic vector (Promega, Madison, WI).

The HPV16 E6 and E7 coding sequences were amplified from the cDNA generated from CaSki total RNA with primers GAAGATCTATGTTTCAGGACCCACAGG and CGGAATTCTTACAGCTGGGTTTCTCTAC for E6 and GAAGATCTATGCATGGAGATACACCTAC and CGGAATTCTTATGGTTTCTGAGAACAG for E7. The PCR products were digested with BglII and EcoRI and inserted into pMSCV-puro, which was digested with the same enzymes.

The short hairpin sequences were designed to target HPV16 E6 and/or E7 expression based on previously published sequences^32^. Oligonucleotides TGCACAGAGCTGCAAACAACTCAAGAGAGTTGTTTGCAGCTCTGTGCTTTTTTC and TCGAGAAAAAAGCACAGAGCTGCAAACAACTCTCTTGAGTTGTTTGCAGCTCTGTGCA for E6 and TGACAGAGCCCATTACAATATCAAGAGATATTGTAATGGGCTCTGTCTTTTTTC and TCGAGAAAAAAGACAGAGCCCATTACAATATCTCTTGATATTGTAATGGGCTCTGTCA for E7 were synthesized by Life Technologies (Shanghai, China), annealed, and inserted into HpaI/XhoI digested pLL3.7 (MIT, Cambridge, MA). All constructs were verified by DNA sequencing (Life Technologies).

The retroviruses and lentiviruses were produced and titered by Biowit Technologies (Shengzheng, China).

### Western Blot

The cells from a 6-well plate were lyzed in 100 μl RIPA buffer (50mM Tris-cl pH 7.4, 150mM NaCl, 1% NP40, 0.25% Na-deoxycholate) including 1 mM PMSF, 1x complete protease inhibitor cocktail (Roche Diagnostics, Indianapolis, IN), and 1x phosphatase inhibitor cocktail (Pierce, Rockford, IL) with 3 cycles of freeze-and-thaw. After 15 min of centrifugation at 10000g at 4°C, the supernatant was collected and the concentration of total protein was measured using a BCA kit (Pierce). An aliquot of 25 μg total protein was resolved in a 10% sodium dodecyl sulfate polyacrylamide gel and blotted onto a polyvinylidene difluoride membrane. The membrane was blocked in 5% nonfat milk in TBST (50 mM Tris-HCl, pH 7.4, 150 mM NaCl, 0.1% Tween 20) at room temperature for 30 min before incubation with either anti-E6 (C1P5, Abcam, Cambridge, MA), GSK3β (ab31826, Abcam), or phsopho-GSK3β (S9) (ab118885, Abcam) antibodies at 4 °C overnight. Next, the membrane was washed 3 times with TBST and incubated with appropriate horseradish peroxidas-conjugated secondary antibodies (Jackson ImmunoResearch Laboratories, West Grove, PA) for 60 min at room temperature. The specific protein bands were visualized using ECL Western Blotting Substrate (Pierce).

### MTT assay

The cells were seeded in 96-well plates a day before being infected with retroviruses or lentiviruses. 48 hr after the virus infection, the cell culture medium was replaced with 100 μl fresh growth medium containing 10 μl of 12 mM 3-(4,5-dimethylthiazol-2-yl)-2,5-diphenyltetrazolium bromide (MTT, Life Technologies) and continued incubation at 37 °C for 4 hr, and then the medium was replaced with 150 μl of DMSO. The plate was protected against light and incubated at room temperature for 10 min with shaking before being read at 490 nm on a plate reader (Molecular Devices, Sunnyvale, CA).

### Cell invasion assay

The cells were trypsinized and adjusted to a concentration of 5 × 10^4^ cells/ml with serum-free medium. 200 μl cells were seeded in the upper chambers of 12-well Transwell plates (BD BioSciences, San Jose, CA) and 600 μl of complete growth medium containing 10% FBS was put into the lower chambers. After culturing for 24 hr at 37 °C, the cells were fixed with methanol for 15 min at room temperature and stained with Giemsa solution for 15 min before rinsing with PBS and being wiped off with Matrigel (BD BioSciences) on the upper surface. The number of migrated cells was counted from 5 random fields (200x).

### Colony formation assay

A single cell suspension was diluted to a concentration of 10^3^ cells/ml with complete growth medium and 100 μl of the cell suspension was mixed with 0.3% agar Noble agar (DIFCO, Detroit, MI) and was seeded onto a layer of solidified agar which was previously prepared in a 24-well cell culture plate (Corning, Tewksbury, MA). The plate was incubated at 37 °C until clones were formed.

### Statistical analysis

All experiments were independently performed in triplicates. The data were expressed as mean ± standard deviation. SPSS 16.0 statistical software was used for data analysis of variance (ANOVA) or independent sample t-test. A P value less than 0.05 was considered statistically significant.

## Additional Information

**How to cite this article**: Ma, C. *et al.* GSK3β mediates the carcinogenic effect of HPV16 in cervical cancer. *Sci. Rep.* **5**, 16555; doi: 10.1038/srep16555 (2015).
